# Gender differences in premature mortality for cardiovascular disease in India, 2017–18

**DOI:** 10.1186/s12889-023-15454-9

**Published:** 2023-03-23

**Authors:** Jhumki Kundu, K. S. James, Babul Hossain, Ruchira Chakraborty

**Affiliations:** International Institute for Population Sciences, 400088 Mumbai, India

**Keywords:** Years of life lost (YLL), CVD, Gender differences, India

## Abstract

**Background:**

The present study tries to provide a comprehensive estimate of gender differences in the years of life lost due to CVD across the major states of India during 2017–18.

**Methods:**

The information on the CVD related data were collected from medical certification of causes of death (MCCD reports, 2018). Apart from this, information from census of India (2001, 2011), SRS (2018) were also used to estimate YLL. To understand the variation in YLL due to CVD at the state level, nine sets of covariates were chosen: share of elderly population, percentage of urban population, literacy rate, health expenditure, social sector expenditure, labour force participation, HDI Score and co-existence of other NCDs such as diabetes, & obesity. The absolute number of YLL and YLL rates were calculated. Further, Pearson’s correlation had been calculated and to understand the effect of explanatory variables on YLL due to CVD, multiple linear regression analysis had been applied.

**Results:**

Men have a higher burden of premature mortality in terms of Years of life lost (YLL) due to CVD than women in India, with pronounced differences at adult ages of 50–54 years and over. The age pattern of YLL rate suggests that the age group 85 + makes the highest contribution to the overall YLL rate due to CVD. YLL rate showed a *J*-shaped relationship with age, starting high at ages below 1 years, dropping to their lowest among children aged 1–4 years, and rising again to highest levels at 85 + years among both men and women. In all the states except Bihar men had higher estimated YLL due to CVD for all ages than women. Among men the YLL due to CVD was higher in Tamil Nadu followed by Madhya Pradesh and Chhattisgarh. On the other hand, the YLL due to CVD among men was lowest in Jharkhand followed by Assam. Similarly, among women the YLL due to CVD was highest in Tamil Nadu followed by Madhya Pradesh and Chhattisgarh. While, the YLL due to CVD among women was lowest in Jharkhand. Irrespective of gender, all factors except state health expenditure were positively linked with YLL due to CVD, i.e., as state health expenditure increases, the years of life lost (YLL) due to CVDs falls. Among all the covariates, the proportion of a state's elderly population emerges as the most significant predictor variable for YLL for CVDs (*r *= 0.42 for men and *r* = 0.50 for women).

**Conclusion:**

YLL due to cardiovascular disease varies among men and women across the states of India. The state-specific findings of gender differences in years of life lost due to CVD may be used to improve policies and programmes in India.

**Supplementary Information:**

The online version contains supplementary material available at 10.1186/s12889-023-15454-9.

## Background

Non-communicable diseases are the major public health and development concerns of the twenty-first century. The non-communicable diseases, which accounts for more than 60% of all deaths globally, includes cardiovascular disease (CVD), various cancers, chronic respiratory disorders, and diabetes [1]. Although NCDs related morbidity and mortality were once prevalent mostly in developed countries, the morbidity and mortality due to NCDs have increased recently in many LMICs [2]. CVDs, such as ischaemic heart disease and stroke, account for 17.7 million deaths and are the leading cause of death [3]. CVDs are set to accelerate even more as a result of demographic shifts, epidemiological transition, and increasing urbanization, which are all linked to an increase in CVD risk factors i.e., smoking, sedentary lifestyle, obesity, hypertension, and hypercholesterolemia [4]. Cardiovascular disease (CVD) also continues to be a leading cause of premature mortality and rising healthcare expenses [5, 6]. By 2025, over 5 million premature CVD deaths in men and 2.8 million in women are projected globally [7], and the disease imposes a significant economic burden on both developed and developing countries [8]. Thus, understanding the impact of CVD related morbidity and mortality is important to understand the health of a country.

The proverb "women get sicker, but men die sooner" is widely supposed, Women have not always outlived males, according to historical demographic figures. However, historical demographic data demonstrate that women have not always outlived men [9]. Men and women are exposed to various social, psychological, and disease risk factors throughout their lives, which contributes significantly to gender differences in morbidity and mortality, including non-communicable disease (NCD). Thus, the seemingly counterintuitive gender disparities in morbidity and death within and across countries are neither universal nor unchanging [10]. Although, evidence from existing literature suggests that men have higher rates of mortality, while women have a higher prevalence of morbidity than men, this situation can be explained by using two broad theories: biological and psychosocial [11, 12]. According to biological theory, genetic and hormonal differences between men and women are the root cause of the majority of health-related outcomes [13]. On the other hand, the psychosocial theory, proposes that the difference is primarily due to the role of women in society and their attitude towards illness. This disparity exists in both developed and developing countries [11].

The long-held beliefs on the health difference between men and women have contributed to the under-recognition of NCDs in women [14]. For instance, there is a widespread belief that women's health is characterized solely by their reproductive capacity [15], despite the fact that chronic diseases, violence, and other injuries account for two-thirds of all deaths and impairments in women [16]. NCDs, particularly CVDs, have traditionally been thought of as exclusively male diseases and women’s non-communicable diseases (NCDs) are only a problem in high-income countries [17]. In fact, the majority of NCD deaths among women occur in low- and middle-income countries, with rates significantly higher than in developed countries [18]. Also, it was considered that NCDs related deaths were prominent exclusively among the elderly [18].

Looking at the most populated country, India, the age-standardized CVD death rate of 272 per 100,000 people, which is much higher than the global average of 235 [1]. According to the WHO's India report, males had higher age-adjusted CVD death rates than women (349 per 100,000 men and 265 per 100,000 women). These rates are two to three times higher than the United States, where men have a mortality rate of 170 per 100,000 and women have a rate of 108 per 100,000 [19]. Until now, Indian studies analysing age-pooled data have mainly reported male dominance in the overall prevalence of CVD. The gender difference in different ages have been reported by only one Indian study to our knowledge, which also showed greater rates in males in all age groups and no sign of reversal, contrary to evidence from high income nations [20].

Saying so, one of the well-established measures to understand the population health is premature mortality. Premature mortality has increased in recent years due to demographic and epidemiological changes that have altered death rates and morbidity patterns across age groups. Although new evidence indicates a decline in infant and child mortality, little is known about India's age and gender distribution of premature deaths. In India, premature mortality, as measured by years of life lost due to CVD, rose by 59%, from 23.2 million in 1990 to 37 million in 2010 [21]. In 2019, CVDs accounted for 38% of the 17 million premature deaths (before age 70) caused by non-communicable illnesses [22]. Despite numerous studies on the trends, differentials, and risk factors for CVD mortality, studies that estimate premature mortality due to CVD in India are scarce [23, 24].It is necessary to count the dead to understand the influence of cardiovascular diseases on mortality, but it is also required to determine how early the deaths occur.

A number of measures have been developed and used to quantify the extent of premature mortality: the potential years of life lost (PYLL), the premature years of potential life lost (PYPLL), the working years of potential life lost (WYPLL) and the valued years of potential life lost (VYPLL). Many new indices have been developed to measure premature mortality such as the DALY (disability adjusted life years), HALY (health adjusted life years) and YLL (years of life lost). Although numerous approaches have been used to correctly analyze and report the prevalence and trends of premature mortality; many of them have severe shortcomings, especially when gathering real early fatalities [25]. The YLL is a versatile, accurate and comprehensive measure of premature mortality. It is able to reflect the mortality patterns dominated by underlying disease processes occurring at early deaths, majority of which could be delayed to older ages or could be prevented with effective public health interventions. It takes into account both the frequency of deaths and the age at which it occurs. YLL (years of life lost) is an important mortality statistic that complements the crude mortality indicators. As a measure of the premature mortality, YLL has the following advantages: (i) it avoids arbitrary age cut-offs, which are never methodologically justifiable, and exclusions of older population groups;(ii) all deaths imply the loss of some potential years of life, which means that deaths at all ages contribute to the quantification of the burden of premature mortality; & (iii) YLL gives more weight to deaths that happen at younger ages [25]. The YLL becomes a key indicator for evaluating and guiding the progress of public health policies and interventions due to its ease of calculation and comprehension. Thus, this research aims to estimate gender differences in years of life lost (YLL) due to cardiovascular diseases (CVDs) across the states of India.

## Data and Methods

### Data source

Three major data sources were used to estimate YLL. These include, the census of India for the years 2001 and 2011, Sample Registration System (SRS), 2018 and medical certification of causes of death Report (MCCD), 2018. The data on age-sex structure of the population of India and its states were taken from the C13 table of the Census of India. The age-specific death rates for the states for the year 2018 (latest available data) were taken from the SRS Statistical Report. Data on cause-specific deaths across the states of India were taken from the Medical Certification of cause of death (MCCD) reports. Although the MCCD report has the limitation of a lack of representativeness of information, we used it due to the unavailability of other sources of state-specific cause of death information in India. Therefore, we used the proportion of CVD deaths rather than the absolute number of CVD deaths from the MCCD reports.

Explanatory variables used to establish a correlation with YLL due to CVDs, were chosen through extensive literature study from different data sources. The share of the elderly population, literacy rate, percent urban and workforce participation rate were calculated from the Census of India, 2011. To adjust the potential chronic disorders, prevalence of obesity and diabetes for 15 to 49 years of women and 15 to 54 for men were taken from the National family health survey (NFHS-4, 2015–16). The data for state-wise social security-related expenditure was taken from RBI report of state finances whereas state-wise health expenditure data was taken from NSSO 75^th^ round report. The data of state-wise human development Index (HDI) score was taken from the ministry of statistics and programme implementation, govt of India, for the year of 2017–18. Gender stratified analysis was carried out in this study.

### Methodology

Estimates of YLL (Years of Life Lost) were used in the analysis. YLL was calculated considering the discounting rate of 3% [26–29].The discounting rate reflects the social preference for a healthy year now rather than in the future. The value of a year of life is generally decreased annually by a fixed percentage. Both the World Bank's Disease Control Priorities study and the Global Burden of Disease (GBD) project used a 3% discount rate, and the US Panel on Cost-Effectiveness in Health and Medicine recently recommended that health economic analyses also use a 3% real discount rate to adjust both costs and health outcomes [30, 31].

- *The absolute number of YLL*

The absolute number of YLL is estimated as:

$$YLL=\frac{N}{r}(1-{e}^{-rL})$$

where N is the number of deaths, L is the life expectancy at the age of death, and r is the discount rate. This metric quantifies the absolute number of YLL due to premature deaths in certain population.

- *Calculation of YLL rates*

YLL rate due to cause *c*, in the population of sex *s* and age *a*, and time *t* can be calculated by the formula:

$$YLL\;rate \left(c,s,a,t\right)=\frac{YLL\left(c,s,a,t\right)}{P(s,a,t)}*100000\;population$$

where YLL (c, s, a, t) is the number of YLL due to cause c, in population of sex s and age a, and period t, P (s, a, t) is the population size at sex s, age a and period t.

YLL rate provides a relative quantification of the magnitude of the effect of diseases, injuries, and risk factors on premature mortality in the population, but it does not account for variations in the population's age distribution [25]. It is useful for the comparison of sex and age groups, for instance, age-sex-specific YLL rates, is helpful.

Estimation of YLL involves following steps-

- *Step 1*: age-sex specific population for 2017 was projected by the exponential growth rate method for the major states of India. The most frequently used mathematical model assumes that population growth will follow an exponential distribution, which is a generalization of the geometric function when time t is considered to be a continuous variable [32].

The exponential growth of population leads to the equation

$$\mathrm{Pt}=\mathrm P0\;\mathrm e^\wedge\mathrm{rt}$$

Where Pt = Population in 2017.

P0 = Population in 2011

r = Exponential rate of population growth as per person per year; and

t = Period in years elapsed from year 0 to t (2017–2011 = 7 years)

- *Step 2*: The age-sex-specific death rate (ASDR) from SRS -2018 was multiplied by the projected 2017 population to get the age-sex-specific numbers of total deaths.
- *Step 3*: The proportion of the CVD deaths from MCCD reports (Medical Certification of Cause of death) were multiplied with the numbers of total deaths from step 2 to obtain the age-sex specific numbers of deaths due to CVDs. The total numbers of deaths and deaths due to CVD, thus obtained, were aggregated into ten age groups: below 1 year, 1–4 years, 5–14 years, 15 -24 years, 25–34 years, 35–44 years, 45–54 years, 55–64 years,65–69 years and 70 years or older.
- *Step 4*: Age-sex-specific life tables for all-cause were constructed for India (2017–18). Then, YLL due to CVD was calculated for the major states of India.

To establish the relationship between the independent [share of elderly population, literacy rate, percent urban, work force participation rate, prevalence of obesity and diabetes, social security related expenditure, Health expenditure & Human Development Index (HDI)] and dependent variable (YLL due to CVD), Pearson’s correlation had been calculated and to understand the effect of explanatory variables on YLL due to CVD, multiple linear regression analysis has been done.

The equation for the regression is as follows,

$$y=a+{\beta }_{1}{X}_{1}+{\beta }_{2}{X}_{2}+{\beta }_{3}{X}_{3}+\dots +{\beta }_{n}{X}_{n}+\in$$

Where, ${\beta }_{1},{\beta }_{2},{\beta }_{3}$ are regression coefficients, shows the effect of ${X}_{1}, {X}_{2},{X}_{3}$ (e.g. share of elderly population, rate of urbanisation, the prevalence of diabetes etc.) on the value of YLL due to CVDs.

### Robustness check of YLL estimation using MCCD data

**Fig. 1:**
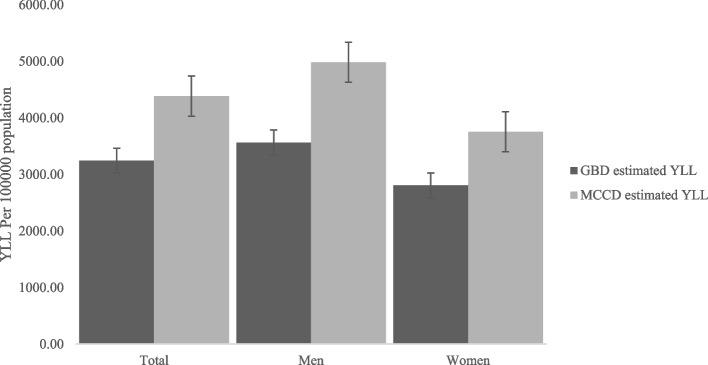
Comparing the YLL estimates between MCCD estimates and GBD estimates for robustness check

The estimated YLL was validated using GBD estimates. The comparison of our estimated YLL vis-à-vis GBD estimates as a strong robustness cheek for our estimates. For instance, Fig. 1 shows the comparison of our estimated YLL to the GBD estimated YLL. Our estimated YLL is lower compared to GBD estimated YLL (Fig. 1). The most notable discrepancies between GBD estimates and our estimated YLL for just a few conditions. We used Medical Certification of cause of death (MCCD) report due to the unavailability of other sources of state-specific cause of death information in India. However, the MCCD report is based primarily on urban hospital deaths (either public or private). Due to incomplete coverage and inadequate quality of data, use of this MCCD data has been compromised. Therefore, our estimated YLL is lower than GBD estimates. Our estimated YLL may lack the representative character in the strict sense, however, it may be sufficient to throw some valuable insights into YLL by CVD in India and its states.

## Results

### Age and gender wise years of life lost (YLL) for India

**Fig. 2:**
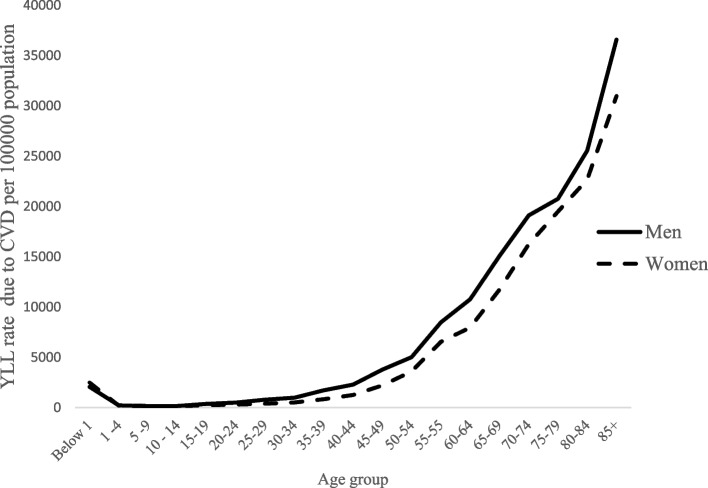
Age -specific YLL rates per 100,000 population by gender, in India,2017–18

Table 1 illustrates the estimated age-specific YLL rates due to CVD among men and women in India. The estimated YLL rate for all ages to be 3565.51 [3347.23–3812.96] among per 100,000 men and 2804.9 [2399.91–2970.82] among per 100,000 women in India (Table 1). The YLL distribution pattern by age and gender can be explored and analysed using age-and-sex-specific YLL rates. Figure 2 shows that age group 60–64, 65–69,70–74,75–79,80–84 and 85 + had the highest YLL rates, which is consistently higher in men compared with women across age groups. YLL rate showed a *J*-shaped relationship with age, starting high at ages below 1 years, dropping to their lowest among children aged 1–4 years, and rising again to highest levels at 85 + years among both men and women. The age pattern of YLL suggests that the age group 85 + makes the highest contribution to the overall YLL rate due to CVD.

**Table 1:** Years of life lost (YLL) rate due to CVD per 100,000 population by gender in India, 2017–18

_INDIA_								
_Age groups_	_**Men**_	_**Women**_	_**Men**_			_**Women**_		
	_**CVD deaths**_		_**YLL rate**_	_**YLL rate 95% CI (LL)**_	_**YLL rate 95% CI (UL)**_	_**YLL rate**_	_**YLL rate 95% CI (LL)**_	_**YLL rate 95% CI (UL)**_
_0–1_	_9185_	_9808_	_2062.35_	_1576.32_	_3658.81_	_2491.41_	_1620.94_	_3666.27_
_1–4_	_3369_	_2710_	_223.23_	_199.45_	_297.12_	_201.64_	_199.77_	_377.96_
_5–9_	_3231_	_3188_	_155.51_	_121.32_	_191.83_	_170.53_	_139.72_	_195.39_
_10–14_	_3877_	_3053_	_168.70_	_132.15_	_194.71_	_149.27_	_101.48_	_152.45_
_15–19_	_9644_	_5333_	_372.30_	_311.88_	_434.07_	_233.54_	_240.45_	_335.31_
_20–24_	_13343_	_7762_	_501.97_	_425.13_	_594.63_	_304.93_	_287.69_	_387.53_
_25–29_	_20488_	_9687_	_792.76_	_742.43_	_941.83_	_395.88_	_370.37_	_497.10_
_30–34_	_23107_	_10739_	_994.53_	_862.58_	_1099.07_	_511.69_	_479.37_	_642.17_
_35–39_	_36395_	_16366_	_1726.07_	_1726.07_	_1570.68_	_841.35_	_820.77_	_1041.11_
_40–44_	_45339_	_22540_	_2294.14_	_2004.87_	_2459.42_	_1260.09_	_1218.51_	_1571.95_
_45–49_	_69531_	_36651_	_3783.00_	_3411.71_	_4197.86_	_2215.73_	_1981.23_	_2498.82_
_50–50_	_82842_	_53264_	_5017.57_	_4320.04_	_5252.73_	_3604.28_	_3287.82_	_4061.36_
_55–59_	_125188_	_90730_	_8477.23_	_7398.99_	_9054.62_	_6543.58_	_5640.72_	_6920.25_
_60–64_	_161352_	_108559_	_10776.12_	_9036.45_	_10892.36_	_7963.11_	_7225.01_	_8657.68_
_65–69_	_186418_	_133201_	_15069.48_	_13509.84_	_16180.65_	_11718.66_	_10598.59_	_12896.54_
_70–74_	_176140_	_151478_	_19109.76_	_16565.94_	_20269.74_	_16242.87_	_14585.57_	_18333.72_
_75–79_	_144870_	_134842_	_20759.90_	_19166.77_	_23494.30_	_19487.90_	_17722.70_	_22394.72_
_80–84_	_117349_	_113732_	_25552.84_	_21334.08_	_25894.26_	_22676.64_	_21056.61_	_27839.13_
_85+_	_116903_	_125120_	_36587.34_	_28939.91_	_36433.21_	_30975.06_	_26375.66_	_33681.06_
_Total_	_1348570_	_1038765_	_3565.51_	_3347.23_	_3812.96_	_2804.9_	_2391.99_	_2970.82_

### Gender difference in YLL rate across the states in India

**Fig. 3:**
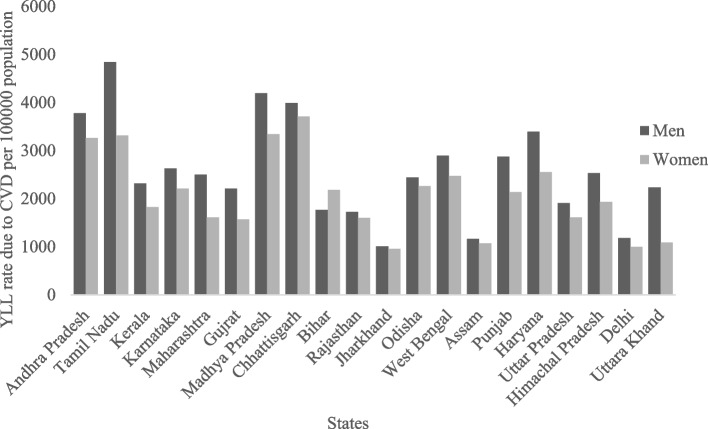
YLL rates per 100,000 population by gender across the major states of India, 2017–18

There were substantial regional variations in the years of life lost (YLL) due to CVD in India. We also observed gender differences in the Years of life lost (YLL) due to CVD across the states in India. In all states except Bihar (1768.76 YLL per 100,000 men vs 2184.03 YLL per 100,000 women), men had higher estimated YLL due to CVD for all ages than women. The gender differences in Years of lost (YLL) due to CVD was higher in Tamil Nadu among the states. Among men the years of life lost (YLL) due to CVD was higher in Tamil Nadu (4842. 51 per 100,000 men) followed by Madhya Pradesh (4192.98 per 100,000 men) and Chhattisgarh (3992.86 per 100,000 men). On the other hand, the YLL due to CVD among men was lowest in Jharkhand (1011.59 per 100,000 men) followed by Assam (1167.13 per 100,000 women) (Fig. 3). Among women the years of life lost due to CVD was also highest in Tamil Nadu (3710.37 per 100,000 women) followed by Madhya Pradesh and Chhattisgarh. On the other hand, the years of life lost (YLL) due to CVD mong women was lowest in Jharkhand (957.68 per 100,000 women) (Fig. 3). The full results for YLLs by gender, and age for the major states were provided in the Supplementary Tables 1.

### Association between years of life lost and correlates by gender

Correlations between the outcome variable of years of life lose (YLL) owing to CVDs and state-specific explanatory factors by gender were shown in Tables 2 and 3. To better understand the variation in YLL at the state level, nine sets of covariates were chosen: the share of the elderly population, the percentage of the urban population, the literacy rate, health expenditure, social sector expenditure, labour force participation, and the coexistence of other NCDs such as diabetes, obesity, and Human development Index (HDI)etc. The effect of explanatory variables on YLL owing to CVD was examined using multiple linear regression analysis (Table 4). The Variance Inflation Factor test was performed to determine if any regression model was multicollinear, and the mean result was found to be between 2.66 to 3.01, suggesting no multicollinearity. All factors included in a regression model may account for the connection between YLL and men (64%) and YLL and women (49%). Irrespective of gender, all factors except state health expenditure were positively linked with YLL due to CVD, i.e., as state health expenditure increases, the years of life lost (YLL) due to CVDs falls. Among all factors, the proportion of a state's elderly population emerges as the most significant predictor variable for YLL for CVDs (r = 0.42 for men and r = 0.50 for women). With rising rates of obesity and diabetes, the incidence of early mortality from CVDs also increased, resulting in a high YLL. The situation was similar for the labour force participation rate, social security spending, urban population share, and HDI score although the association is not as strong.

**Table 2:** Association between the years of life lost (YLL) due to CVD among men and socio-economic and health related variables, India, 2017–18

	Years of life lost (YLL) due to CVD among men	Obesity	Diabetes	Share of elderly population	Percentage of male population in Urban area	Male literacy Rate	Health expenditure	Social sector expenditure	Labour force Participation	Human development Index (HDI)
Years of life lost (YLL) due to CVD	1									
Obesity	0.25	1								
Diabetes	0.19	0.46	1							
The share of elderly population	0.42	0.64	0.60	1						
Percentage of male population in urban area	0.13	0.56	0.20	0.08	1					
Male literacy rate	0.08	0.61	0.30	0.34	0.73	1				
Health expenditure	-0.22	0.35	-0.31	0.61	0.47	0.35	1			
Social sector expenditure	0.39	0.25	0.07	0.21	0.13	0.12	0.28	1		
Labour force participation	0.24	-0.14	0.02	-0.17	0.01	-0.17	-0.14	-0.20	1	
Human development Index (HDI)	0.31	0.83	0.27	0.38	0.65	0.83	0.47	-0.42	0.14	1

**Table 3:** Association between the years of life lost (YLL) due to CVD among women and socio-economic and health rated variables, India, 2017–18

	**Years of life lost (YLL) due to CVD among women**	**Obesity**	**Diabetes**	**Percentage of elderly population**	**Percentage of Female population in urban area**	**Female literacy rate**	**Health expenditure**	**Social sector expenditure**	**Labour force Participation**	**Human Development Index (HDI)**
Years of life lost (YLL) due to CVD among women	1									
Obesity	0.35	1								
Diabetes	0.24	0.62	1							
The share of elderly population	0.50	0.69	0.62	1						
Percentage of female population in urban area	0.16	0.55	0.28	0.31	1					
Female literacy rate	0.13	0.68	0.63	0.58	0.62	1				
Health_ expenditure	-0.31	0.34	-0.15	0.41	0.47	0.32	1			
Social sector expenditure	0.35	0.23	0.07	0.10	0.13	0.22	0.28	1		
Labour force participation	0.32	-0.28	0.12	-0.21	0.18	-0.23	-0.19	-0.01	1	
Human Development Index (HDI)	0.21	0.79	0.39	0.53	0.64	0.88	0.47	-0.42	0.29	1

**Table 4:** Regression analysis showing the effect of selected socio-economic and health-related variables on YLL Due to CVD, India, 2017–18

Explanatory variables	Male		Female	
	**Coefficient**	**Confidence Interval**	**Coefficient**	**Confidence Interval**
Share of elderly population	4.40	1.31, 7.49	1.47	-1.82,4.76
Share of urban population	1.21	-0.01, 2.43	0.26	-0.89,1.40
Literacy rate	-8.74	-21, 3.53	-3.01	-9.67,3.64
State-health expenditure	-1.37	-3.41, 0.68	0.56	-1.92,3.05
Obesity	-0.30	-1.29, 0.67	0.01	-1.85,1.88
Diabetes	-1.01	-2.5, 0.47	-0.86	-2.76,1.08
Social sector expenditure	0.05	-0.66, 0.77	0.07	-0.86,0.96
Labour force participation	3.06	-3.31, 9.48	0.10	-0.62,0.86
HDI	3.47	-7.09,15.04	2.06	-11.48,15.56
Constant	12.30	-19.8, 44.40	7.18	-6.48,22.48
**R square**	**0.64**		**0.49**	

## Discussion

The present study provides the estimate of years of life lost (YLL) due to cardiovascular diseases (CVDs) by gender across the states of India (2017–18). The estimated YLL rate due to CVD was higher among men than women in India. According to the current study, YLL rates were higher in the age groups 60 and older up to 85 + due to CVD, with 85 + making the largest contribution. Men have a higher burden of premature mortality in terms of Years of life lost (YLL) due to CVD than women, with pronounced differences at adult ages of 40–44 years and above. We observed a remarkable gender differences in YLLs across the states of India. In all states except Bihar, men had higher estimated YLL due to CVD than women. The gender differences in Years of lost (YLL) due to CVD was higher in Tamil Nadu among the states. Irrespective of gender, all the covariates were positively correlated with YLL due to CVD except health expenditure. Among all the covariates, the proportion of a state's elderly population emerges as the most significant predictor variable for YLL for CVDs.

Men in India lost more years of life (YLL) than women in terms of years lost to CVD, supporting earlier findings that men had significantly higher age-standardized rates of cardiovascular disease (CVD) [33]. This finding is consistent with Zhang et al.’s (2021) [34] study, which found that globally men had a premature death rate from cardiovascular diseases was nearly 35.6% higher than that of women. However, the current study's finding is contradicting with Jie et al.’s (2014) study, which found that women lost more life expectancy (LE) than men owing to CVD in China [35]. Around 5% of WHO member states (eight countries in 2016, including Ghana, Mali, Sao Tome and Principe, Zimbabwe, Bhutan, Republic of the Congo, and Nigeria) also had a higher premature death rate from CVDs in women than men [34].

The present study found that Years of life lost (YLL) due to CVD varied significantly between Indian states, corroborating an earlier study that found that regardless of the country's level of development, stage of epidemiological transition, or age composition, cardiovascular mortality is not uniformly distributed across the country [36–42]. Jha (2009) also revealed that there are large regional differences in cardiovascular mortality in India among both men and women [43]. A previous study also highlighted that CVDs are geographically dependent and vary among areas and states due to differences in lifestyle and eating habits among residents of different states [44]. Our study revealed that the years of life lost (YLL) due to cardiovascular disease (CVD) were higher in the southern states of Tamil Nadu and the central states of Madhya Pradesh and lower in the eastern states of Jharkhand and the north-eastern states of Assam. Kaur et al. (2019) [45] in their study also revealed that Ischaemic heart disease was the leading cause of years of life lost due to premature mortality in Tamil Nadu in 2016. This might be as a result of Tamil Nadu being one of the southern Indian states with an advanced level of epidemiological transition [45]. The findings of the present study also corroborate the finding of an earlier study suggesting that there is considerable geographic variation in CVD mortality in India, with less developed regions, such as eastern and north-eastern states with low Human Development indices, experiencing lower proportionate CVD mortality than more developed states in the south and west [46].

Our study demonstrates that health expenditure is negatively correlated with Years of life (YLL) due to CVD. It can also be pointed out that with State’s intervention through increasing health expenditure the years of life lost due to CVD can be reduced [47]. This finding is further supported by the study of Farahani et al. (2010) which showed that a 10% increase in public spending on health in India decreases the average probability of death by about 2%, with effects mainly on the young, the elderly, and women [48]. It is also noted from the present study that with rising rates of obesity and diabetes, the incidence of early mortality from CVDs also increased, resulting in a high YLL. Compared to age, sex, and county-matched controls, patients with type 2 diabetes (T2D) had a 15% higher risk of premature all-cause mortality and a 14% higher risk of cardiovascular death, however, the risk varied depending on the patient's age and glycemic control level [49]. The finding of the present study revealed that there is a positive association of obesity prevalence and Years of life lost due to CVD among women which is further supported by the study of Dikaiou et al. (2021) who stated that there is a significant increase in the risk for early CVD death in overweight young women, with a marked increase in obese women [50].

## Strengths and Limitation

This is the first ever study that provides estimates of Years of life lost (YLL) due to CVD by gender across the states of India. Some studies used GBD data to estimate YLL but GBD data and the modelling techniques are not in the public domain and hence have not been reproduced in other studies, However, it is not possible to determine how these data were used because changes in model specifications and variable data inputs are not public [51], leading to an inability to understand trends or to compare them with estimates using other methods.

Like every other data-based research this study is also bounded by some limitations; First, this study was unable to assess the YLL of CVDs in all Indian states due to a lack of comprehensive data, and hence 20 major states were selected for the present study. Second**,** quality of YLL will depend ultimately on the quality of mortality statistics, assessed by the level of registration coverage, timeliness, completeness and accuracy of underlying causes of death diagnosis and coding. The information on CVD deaths across the states of India was obtained for this study from the Medical Certification of Cause of Death (MCCD) reports in order to estimate the YLL due to CVD. MCCD is the only key source of cause specific death statistics across states in India. However the MCCD report is based on hospital deaths (public or private) that have received medical certification and that too mostly from urban areas, so at the national level, the reliability and quality of this information is a major issue. Therefore, our estimated YLL may lack the representative character in the strict sense, but it may be sufficient to throw some valuable insights into YLL by CVD in India and its states. Third**,** the observed state-specific years of life lost (YLL) due to cardiovascular disease were puzzling to explain, and the relationships between state-specific variables and years of life lost (YLL) due to cardiovascular disease may not have been evaluated conclusively due to data availability constraints.

## Conclusion

From the above findings it is clear that the years of life lost (YLL) due to CVD differs by gender across the states of India. The state-specific findings of gender differences in years of life lost due to CVD may be used to improve policies and programmes, allowing for more persuasive planning of cardiovascular disease prevention and treatment in each state of India, hence advancing progress toward meeting national and global objectives for cardiovascular disease reduction.

## Supplementary Information

**Additional file 1: Supplementary Tables 1.** YLL rates by 5 years age groups in selected states in India.

## Data Availability

Data has been taken from the multiple sources and publicly avavilable. The census of India-https://censusindia.gov.in Sample Registration System (SRS)-https://censusindia.gov.in/census.website/node/294 Medical certification of causes of death Report-https://censusindia.gov.in/census.website/data/MCCDREP National Family health survey (NFHS 4) report-http://rchiips.org/nfhs/NFHS-4Report.shtml RBI report-https://m.rbi.org.in//Scripts/AnnualPublications.aspx?head=State%20Finances%20:%20A%20Study%20of%20Budgets NSSO 75^th^ round report-https://www.thehinducentre.com/resources/article30980139.ece/binary/KI_Health_75th_Final_compressed.pdf Ministry of statistics and programme implementation, govt of India-https://www.indiastat.com/table/economy/state-wise-human-development-index-hdi-scores-dime/1423064. All data generated during this current study are included in this manuscript.

## Abbreviations

YLL: Years of life Lost
ASDR: Age-specific Death rate
MCCD: Medical Certification of Cause of Death
SRS: Sample Registration System
NCD: Non-communicable Disease
CAD: Coronary artery disease
