# Supplementary material for: Gender differences in premature mortality for cardiovascular disease in India, 2017–18
Source: BMC Public Health. 2023 Mar 23;23:547. doi: 10.1186/s12889-023-15454-9 (PMC10035272; doi:10.1186/s12889-023-15454-9)
Supplement: Supplementary file 1 — Additional file 1: Supplementary Tables 1. YLL rates by 5 years age groups in selected states in India. [file 12889_2023_15454_MOESM1_ESM.docx]

| Supplementary tables 1: YLL rates by 5 years age groups in selected states in India. | | | | | |
| --- | --- | --- | --- | --- | --- |
| Andhra Pradesh | | | **Tamil Nadu** | | |
| Age groups | **YLL rate per 100000 population** | | **YLL rate per 100000 population** | | |
|  | **Male** | **Female** | **Male** | | **Female** |
| 0-1 | 4353.00 | 3208.41 | 2664.33 | | 2425.43 |
| 1 -4 | 632.81 | 481.86 | 421.07 | | 444.15 |
| 5 -9 | 211.37 | 227.69 | 386.60 | | 187.76 |
| 10 - 14 | 309.12 | 278.31 | 226.16 | | 122.40 |
| 15-19 | 393.05 | 340.38 | 998.57 | | 381.73 |
| 20-24 | 632.36 | 494.69 | 1489.72 | | 665.75 |
| 25-29 | 886.21 | 556.44 | 1446.01 | | 797.15 |
| 30-34 | 1557.19 | 1182.13 | 1651.53 | | 931.42 |
| 35-39 | 2258.05 | 1150.34 | 2927.61 | | 1214.52 |
| 40-44 | 3287.14 | 1710.82 | 4675.86 | | 1800.69 |
| 45-49 | 6173.92 | 4782.54 | 6297.50 | | 2217.07 |
| 50-54 | 7262.07 | 5268.81 | 9953.72 | | 5033.51 |
| 55-59 | 10194.04 | 8654.70 | 12336.02 | | 8115.15 |
| 60-64 | 13693.86 | 12994.16 | 12999.17 | | 10934.42 |
| 65-69 | 15353.90 | 18010.36 | 21887.97 | | 17232.38 |
| 70-74 | 18274.34 | 22761.56 | 22498.00 | | 22463.99 |
| 75-79 | 23923.22 | 27223.29 | 27368.74 | | 26205.60 |
| 80-84 | 28403.54 | 27226.96 | 28099.85 | | 21583.09 |
| 85+ | 23629.07 | 27011.84 | 24861.04 | | 35581.52 |
| Total | **3777.07** | **3262.59** | **4842.51** | | **3316.58** |
| Kerala | | | **Karnataka** | | |
| Age groups | **YLL rate per 100000 population** | | **YLL rate per 100000 population** | | |
|  | **Male** | **Female** | **Male** | | **Female** |
| 0-1 | 0.00 | 0.00 | 10.69 | | 21.51 |
| 1 -4 | 0.00 | 36.56 | 24.65 | | 19.44 |
| 5 -9 | 15.64 | 84.48 | 29.05 | | 15.35 |
| 10 - 14 | 30.49 | 20.65 | 14.16 | | 22.51 |
| 15-19 | 140.46 | 83.09 | 170.36 | | 150.70 |
| 20-24 | 112.99 | 120.76 | 226.11 | | 229.45 |
| 25-29 | 367.65 | 98.81 | 1118.87 | | 484.02 |
| 30-34 | 262.44 | 165.31 | 798.68 | | 462.72 |
| 35-39 | 696.09 | 169.21 | 1697.38 | | 781.61 |
| 40-44 | 1258.91 | 475.35 | 2174.43 | | 1052.10 |
| 45-49 | 1340.49 | 792.80 | 3371.51 | | 2169.90 |
| 50-54 | 2460.48 | 1212.58 | 4567.68 | | 3284.29 |
| 55-59 | 4424.32 | 2142.59 | 7665.44 | | 8455.10 |
| 60-64 | 5624.65 | 3987.60 | 12679.17 | | 8181.87 |
| 65-69 | 11854.47 | 6965.09 | 16402.17 | | 12522.85 |
| 70-74 | 16386.17 | 11164.63 | 28814.55 | | 22660.14 |
| 75-79 | 21126.81 | 16529.38 | 22481.81 | | 21893.22 |
| 80-84 | 26803.34 | 22170.57 | 24598.48 | | 30682.61 |
| 85+ | 47238.75 | 40488.35 | 34056.12 | | 51191.31 |
| Total | **2319.08** | **1825.19** | **2633.60** | | **2206.46** |
| Maharashtra | | | **Gujrat** | | |
| Age groups | **YLL rate per 100000 population** | | **YLL rate per 100000 population** | | |
|  | Male | **Female** | **Male** | | **Female** |
| 0-1 | 535.46 | 600.70 | 3955.60 | | 3531.68 |
| 1 -5 | 102.66 | 137.50 | 118.59 | | 139.15 |
| 5 -10 | 79.20 | 84.19 | 250.82 | | 243.82 |
| 10 - 15 | 102.96 | 61.75 | 97.82 | | 143.05 |
| 15-20 | 165.58 | 38.29 | 521.37 | | 250.13 |
| 20-25 | 319.68 | 111.31 | 950.64 | | 484.71 |
| 25-30 | 562.74 | 280.95 | 566.66 | | 337.88 |
| 30-35 | 841.66 | 387.96 | 755.07 | | 599.88 |
| 35-40 | 1877.22 | 448.02 | 1383.28 | | 904.72 |
| 40-45 | 2352.05 | 991.60 | 2165.14 | | 1376.67 |
| 45-50 | 3866.14 | 1610.10 | 3934.10 | | 2489.54 |
| 50-55 | 5390.94 | 2064.27 | 6200.75 | | 4375.83 |
| 55-60 | 7908.55 | 4319.88 | 8506.28 | | 5671.89 |
| 60-65 | 11594.05 | 5820.64 | 10043.38 | | 7065.88 |
| 65-70 | 12790.36 | 10909.34 | 8589.89 | | 4950.23 |
| 70-75 | 14518.03 | 11739.20 | 11210.57 | | 6264.80 |
| 75-80 | 17954.98 | 15360.28 | 10583.55 | | 6695.74 |
| 80-85 | 20827.06 | 22732.21 | 14837.18 | | 9389.62 |
| 85+ | 24801.79 | 24308.23 | 21024.26 | | 10014.66 |
| Total | **2498.81** | **1612.58** | **2208.27** | | **1571.37** |
| Madhya Pradesh | | | **Chhattisgarh** | | |
| Age groups | **YLL rate per 100000 population** | | **YLL rate per 100000 population** | | |
|  | **Male** | **Female** | **Male** | | **Female** |
| 0-1 | 369.98 | 277.53 | 656.35 | | 7160.13 |
| 1 -4 | 13.32 | 68.63 | 623.58 | | 678.42 |
| 5 -9 | 73.98 | 98.94 | 607.99 | | 499.65 |
| 10 - 14 | 103.04 | 32.25 | 930.10 | | 61.07 |
| 15-19 | 681.84 | 316.26 | 1077.40 | | 964.87 |
| 20-24 | 708.82 | 268.12 | 1725.36 | | 600.99 |
| 25-29 | 1917.73 | 855.26 | 1172.97 | | 1125.88 |
| 30-34 | 2074.15 | 891.96 | 3890.63 | | 1582.85 |
| 35-39 | 3961.32 | 2109.16 | 2543.73 | | 1919.01 |
| 40-44 | 4305.79 | 2390.82 | 6374.26 | | 4059.80 |
| 45-49 | 6764.11 | 4359.07 | 4952.40 | | 4756.98 |
| 50-54 | 7587.33 | 6979.71 | 14856.86 | | 7543.88 |
| 55-59 | 18664.38 | 12456.66 | 15065.57 | | 11954.82 |
| 60-64 | 21318.21 | 12757.36 | 23377.62 | | 13250.45 |
| 65-69 | 26544.73 | 23900.30 | 38419.44 | | 18828.67 |
| 70-74 | 33226.58 | 28975.12 | 49136.06 | | 25381.36 |
| 75-79 | 30993.69 | 43420.83 | 43399.10 | | 40039.15 |
| 80-84 | 42855.87 | 48685.51 | 71877.13 | | 65751.87 |
| 85+ | 74873.89 | 74790.08 | 3992.86 | | 47250.38 |
| Total | **4192.98** | **3340.05** | **3992.86** | | **3710.37** |
| Bihar | | | **Rajasthan** | | |
| Age groups | **YLL rate per 100000 population** | | **YLL rate per 100000 population** | | |
|  | **Male** | **Female** | **Male** | | **Female** |
| 0-1 | 3404.92 | 7321.49 | 697.85 | | 861.70 |
| 1 -4 | 451.20 | 368.16 | 254.74 | | 177.26 |
| 5 -9 | 213.73 | 263.75 | 174.17 | | 225.13 |
| 10 - 14 | 243.12 | 386.85 | 203.77 | | 256.83 |
| 15-19 | 151.18 | 175.02 | 266.02 | | 299.54 |
| 20-24 | 194.58 | 314.94 | 449.39 | | 354.72 |
| 25-29 | 570.89 | 412.92 | 539.95 | | 254.47 |
| 30-34 | 737.42 | 701.78 | 513.91 | | 451.79 |
| 35-39 | 1125.18 | 819.05 | 1148.30 | | 569.02 |
| 40-44 | 1205.55 | 821.75 | 1818.73 | | 778.75 |
| 45-49 | 2054.10 | 1460.58 | 2423.32 | | 2025.94 |
| 50-54 | 2156.10 | 2044.88 | 3976.17 | | 3197.81 |
| 55-59 | 5852.12 | 7479.13 | 7728.25 | | 5621.09 |
| 60-64 | 8593.99 | 12793.63 | 7846.31 | | 6849.44 |
| 65-69 | 13618.66 | 13761.60 | 13789.13 | | 9053.72 |
| 70-74 | 21337.12 | 35802.59 | 14625.24 | | 12380.63 |
| 75-79 | 25221.92 | 32572.09 | 15628.48 | | 13467.77 |
| 80-84 | 39510.94 | 48568.33 | 19842.34 | | 24803.32 |
| 85+ | 32057.49 | 43013.06 | 31639.53 | | 33617.94 |
| Total | **1768.76** | **2184.03** | **1725.75** | | **1599.79** |
| Jharkhand | | | **Odisha** | | |
| Age groups | **YLL rate per 100000 population** | | **YLL rate per 100000 population** | | |
|  | **Male** | **Female** | **Male** | | **Female** |
| 0-1 | 0 | 0 | 0 | | 0 |
| 1 -4 | 0 | 0 | 282.8 | | 205.9 |
| 5 -9 | 0 | 0 | 167.4 | | 173.6 |
| 10 - 14 | 0 | 0 | 256.4 | | 121.2 |
| 15-19 | 0 | 0 | 205.7 | | 285.1 |
| 20-24 | 0 | 0 | 330.9 | | 303.9 |
| 25-29 | 796.15 | 18.44 | 757.0 | | 243.6 |
| 30-34 | 1089.28 | 22.44 | 995.0 | | 532.3 |
| 35-39 | 827.83 | 847.98 | 1281.8 | | 1070.0 |
| 40-44 | 1380.17 | 1671.68 | 2201.9 | | 1821.7 |
| 45-49 | 2258.44 | 1464.76 | 2765.3 | | 2070.0 |
| 50-54 | 2648.17 | 2660.41 | 3428.9 | | 3454.8 |
| 55-59 | 4082.86 | 4158.24 | 7104.7 | | 7026.4 |
| 60-64 | 4186.55 | 6235.68 | 8570.7 | | 9058.4 |
| 65-69 | 5974.12 | 5719.82 | 13943.7 | | 14541.3 |
| 70-74 | 7593.51 | 6737.06 | 16901.0 | | 15613.9 |
| 75-79 | 11683.54 | 12975.85 | 18148.2 | | 20927.8 |
| 80-84 | 11095.97 | 14773.48 | 15005.0 | | 20281.7 |
| 85+ | 18474.01 | 20143.91 | 18591.0 | | 20375.9 |
| Total | **1011.59** | **957.68** | **2442.7** | | **2261.5** |
| West Bengal | | | **Assam** | | |
| Age groups | **YLL rate per 100000 population** | | **YLL rate per 100000 population** | | |
|  | **Male** | **Female** | **Male** | | **Female** |
| 0-1 | 1166.86 | 1615.64 | 0 | | 0 |
| 1 -4 | 257.51 | 141.54 | 0 | | 0 |
| 5 -9 | 49.01 | 61.89 | 0 | | 0 |
| 10 - 14 | 95.57 | 60.52 | 0 | | 0 |
| 15-19 | 202.98 | 279.90 | 490.08 | | 415.29 |
| 20-24 | 251.93 | 180.80 | 417.43 | | 201.19 |
| 25-29 | 529.12 | 438.66 | 278.98 | | 686.29 |
| 30-34 | 658.56 | 372.77 | 531.05 | | 426.46 |
| 35-39 | 1321.14 | 587.90 | 1109.09 | | 1602.25 |
| 40-44 | 1427.81 | 1101.01 | 1674.68 | | 1235.58 |
| 45-49 | 3793.78 | 2096.48 | 2818.90 | | 2341.94 |
| 50-54 | 4848.83 | 4721.66 | 2748.53 | | 3172.78 |
| 55-59 | 8396.55 | 8442.57 | 4019.69 | | 3824.61 |
| 60-64 | 13140.31 | 10451.33 | 4641.25 | | 4256.33 |
| 65-69 | 18478.72 | 16449.51 | 6482.07 | | 6435.92 |
| 70-74 | 21408.35 | 19638.39 | 5766.81 | | 4759.12 |
| 75-79 | 28385.74 | 27558.63 | 6247.17 | | 4803.95 |
| 80-84 | 30711.02 | 31276.53 | 9382.27 | | 9323.49 |
| 85+ | 36065.00 | 39108.57 | 8975.40 | | 8301.68 |
| Total | **2897.14** | **2473.46** | **1167.13** | | **1069.11** |
| Uttar Pradesh | | | **Himachal Pradesh** | | |
| Age groups | **YLL rate per 100000 population** | | **YLL rate per 100000 population** | | |
|  | **Male** | **Female** | **Male** | | **Female** |
| 0-1 | 1302.23 | 2069.62 | 2556.62 | | 2405.07 |
| 1 -4 | 112.45 | 56.97 | 451.44 | | 951.63 |
| 5 -9 | 146.10 | 242.23 | 70.81 | | 175.24 |
| 10 - 14 | 213.68 | 207.26 | 103.56 | | 171.35 |
| 15-19 | 496.74 | 206.61 | 179.47 | | 293.36 |
| 20-24 | 539.45 | 314.59 | 346.48 | | 56.85 |
| 25-29 | 689.17 | 237.92 | 786.57 | | 128.53 |
| 30-34 | 983.90 | 284.31 | 484.41 | | 245.75 |
| 35-39 | 1423.12 | 719.92 | 1047.66 | | 449.22 |
| 40-44 | 2377.46 | 1251.96 | 976.08 | | 883.36 |
| 45-49 | 2558.85 | 2432.91 | 2210.50 | | 1081.02 |
| 50-54 | 3777.81 | 4758.74 | 2869.98 | | 1963.42 |
| 55-59 | 9100.97 | 7263.11 | 6066.59 | | 3817.07 |
| 60-64 | 10010.01 | 7357.48 | 6470.21 | | 5331.96 |
| 65-69 | 13581.48 | 10562.81 | 12039.49 | | 6296.50 |
| 70-74 | 16788.06 | 14766.93 | 16579.52 | | 21714.43 |
| 75-79 | 14228.18 | 13507.18 | 29480.94 | | 14830.92 |
| 80-84 | 21003.86 | 22281.96 | 26465.58 | | 12330.44 |
| 85+ | 25407.29 | 22920.12 | 25070.52 | | 30612.77 |
| Total | **1909.43** | **1613.18** | **2528.73** | | **1932.81** |
| Punjab | | | **Haryana** | | |
| Age groups | **YLL rate per 100000 population** | | **YLL rate per 100000 population** | | |
|  | **Male** | **Female** | **Male** | | **Female** |
| 0-1 | 0 | 0 | 20195.49 | | 19693.27 |
| 1 -4 | 291.01 | 133.00 | 498.06 | | 1491.09 |
| 5 -9 | 82.22 | 230.89 | 386.07 | | 346.41 |
| 10 - 14 | 200.41 | 129.02 | 250.94 | | 338.73 |
| 15-19 | 457.60 | 227.66 | 613.88 | | 613.03 |
| 20-24 | 834.37 | 624.97 | 888.87 | | 857.97 |
| 25-29 | 945.69 | 476.11 | 1268.12 | | 710.99 |
| 30-34 | 1500.14 | 682.74 | 2011.62 | | 556.13 |
| 35-39 | 2809.63 | 1083.34 | 2663.14 | | 959.92 |
| 40-44 | 2675.84 | 1594.12 | 3163.51 | | 1452.02 |
| 45-49 | 5756.71 | 1947.31 | 5631.39 | | 2554.09 |
| 50-54 | 5752.53 | 3650.95 | 7346.76 | | 4493.97 |
| 55-59 | 8486.94 | 5967.06 | 7550.82 | | 4086.21 |
| 60-64 | 8958.93 | 6409.30 | 10951.69 | | 6694.41 |
| 65-69 | 11660.35 | 10993.61 | 20189.37 | | 11474.88 |
| 70-74 | 13160.40 | 11035.42 | 25879.89 | | 18652.06 |
| 75-79 | 12500.52 | 15351.85 | 20380.70 | | 25453.60 |
| 80-84 | 12294.19 | 10842.67 | 25687.85 | | 27928.12 |
| 85+ | 21272.00 | 23609.98 | 37259.01 | | 25158.85 |
| Total | **2874.89** | **2138.46** | **3393.00** | | **2550.30** |
| Delhi | | | **Uttarakhand** | | |
| Age groups | **YLL rate per 100000 population** | | **YLL rate per 100000 population** | | |
|  | Male | Female | Male | Female | |
| 0-1 | 1805.07 | 1375.62 | 757.55 | 304.31 | |
| 1 -4 | 375.07 | 316.33 | 21.92 | 29.78 | |
| 5 -9 | 102.17 | 78.89 | 69.67 | 111.16 | |
| 10 - 14 | 124.52 | 38.57 | 84.91 | 86.96 | |
| 15-19 | 270.07 | 322.91 | 124.44 | 113.64 | |
| 20-24 | 186.21 | 208.58 | 240.25 | 357.85 | |
| 25-29 | 416.32 | 229.25 | 673.33 | 301.07 | |
| 30-34 | 356.62 | 394.49 | 640.86 | 340.15 | |
| 35-39 | 922.80 | 476.55 | 1410.12 | 737.03 | |
| 40-44 | 988.72 | 892.47 | 1989.43 | 542.26 | |
| 45-49 | 1674.42 | 1109.13 | 4028.02 | 1038.34 | |
| 50-54 | 2846.72 | 2110.41 | 4951.82 | 1499.05 | |
| 55-59 | 3349.21 | 3013.94 | 8241.47 | 3139.52 | |
| 60-64 | 5647.05 | 4599.40 | 7253.08 | 3797.12 | |
| 65-69 | 6520.81 | 7455.54 | 23825.93 | 8886.82 | |
| 70-74 | 9595.56 | 7927.94 | 15373.68 | 8753.59 | |
| 75-79 | 12948.89 | 9779.39 | 28187.54 | 12577.66 | |
| 80-84 | 10607.53 | 10343.99 | 20852.41 | 7980.95 | |
| 85+ | 47303.29 | 14045.93 | 29254.65 | 9022.18 | |
| Total | **1180.05** | **997.13** | **2237.59** | **1084.68** | |
